# Bridging the Translational Gap in Artificial Intelligence for Otology and Neurotology: Clinical Readiness, Limitations, and Research Priorities

**DOI:** 10.3390/medicina62101895

**Published:** 2026-09-30

**Authors:** Pasquale Viola, Teodoro Aragona, Pietro De Luca, Federico Maria Gioacchini, Alfonso Scarpa, Simona Carvelli, Claudio Petrolo, Roberta Mussari, Giuseppe Chiarella

**Affiliations:** 1 Unit of Audiology, Phoniatrics and Vestibology, Regional Centre for Cochlear Implants and ENT Diseases, Department of Experimental and Clinical Medicine, Magna Graecia University, 88100 Catanzaro, Italy; pasqualeviola@unicz.it (P.V.);; 2 Department of Otorhinolaryngology and Maxillofacial Surgery, IRCCS Research Hospital Casa Sollievo della Sofferenza, San Giovanni Rotondo, 71013 Foggia, Italy; 3 Otolaryngology Department, San Giovanni-Addolorata Hospital, 00184 Rome, Italy; 4 Department of Clinical and Molecular Sciences, Polytechnic University of Marche, 60126 Ancona, Italy; 5 Department of Medicine and Surgery, University of Salerno, 84081 Baronissi, Italy

**Keywords:** artificial intelligence, otology, neurotology, clinical translation, external validation, hearing loss, vestibular disorders, cochlear implantation, medical imaging, digital health

## Abstract

*Background and Objectives*: Artificial intelligence (AI) has generated numerous proof-of-concept applications in otology and neurotology, but relatively few have progressed to routine clinical use. This narrative review evaluates not only what AI can do across audiovestibular medicine, but also how close individual applications are to clinical translation and which barriers continue to limit implementation. *Materials and Methods*: PubMed/MEDLINE, Scopus, and Web of Science were searched from database inception to 31 July 2026 using combinations of terms related to AI, machine learning, deep learning, hearing loss, cochlear implantation, tinnitus, vestibular disorders, nystagmus, temporal bone imaging, digital phenotyping, rehabilitation, and robotic surgery. Reference lists of relevant publications were also screened. Evidence was synthesized thematically and interpreted according to task maturity, external validation, clinical utility, workflow integration, and patient-safety requirements. *Results*: The most mature applications are narrow, well-defined tasks involving image or signal classification and anatomical segmentation, including audiogram pattern recognition, automated nystagmus extraction, temporal-bone segmentation, and vestibular schwannoma volumetry. By contrast, multimodal prognostic models, AI-guided treatment selection, digital phenotyping, autonomous decision support, and robotic or intraoperative systems remain less clinically established. Across domains, translation is constrained by small retrospective datasets, center- and device-specific acquisition patterns, inconsistent reference standards, limited external and prospective validation, inadequate reporting of calibration and failure modes, and scarce evidence of improved patient outcomes or workflow efficiency. *Conclusions*: The principal challenge for AI in otology and neurotology is no longer technical feasibility, but demonstration of generalizable clinical value. Future research should prioritize representative multicenter data, independent validation, prospective impact studies, clinically meaningful comparators, interoperability, explainability, fairness, and post-deployment surveillance. AI should be implemented as an auditable, human-supervised technology that strengthens specialist judgment and patient-centered care.

## 1. Introduction

Artificial intelligence (AI) is increasingly applied to audiograms, speech-perception scores, otoacoustic emissions, auditory evoked potentials, vestibulo-ocular recordings, posturography, computed tomography (CT), magnetic resonance imaging (MRI), device logs, patient-reported outcomes, and wearable-sensor data [1,2,3,4,5,6,7]. Machine learning (ML) and deep learning (DL) can identify complex associations within these data and support classification, segmentation, prediction, and decision-support tasks. The technical feasibility of such approaches is now well established across several areas of otology and neurotology. The clinically important issue is different. Hearing loss, tinnitus, and vestibular disorders are heterogeneous conditions in which diagnostic labels, disease severity, device characteristics, acquisition protocols, and treatment pathways vary substantially across centers and populations [8,9,10,11]. An algorithm may therefore perform well within a development dataset while failing when transferred to another clinic, scanner, audiometer, video-oculography platform, language, or patient group. Technical accuracy alone does not establish clinical usefulness. Several recent reviews have cataloged AI applications in audiology, otology, and neurotology [4,5,7]. The remaining gap is translational: which applications have progressed beyond proof of concept, which evidence is still missing, and what methodological and regulatory requirements should precede routine use? Many published studies remain retrospective, single-center, narrowly task-specific, and dependent on internal validation [12,13,14]. Few demonstrate improved decisions, patient outcomes, safety, accessibility, or efficiency in real-world workflows. Accordingly, this narrative review is organized around clinical readiness rather than technological novelty alone. It examines AI applications in hearing assessment and rehabilitation, cochlear implantation, vestibular medicine, tinnitus, otological imaging, surgery, and digital monitoring, and appraises each domain according to data quality, validation status, workflow integration, clinical utility, and patient-safety implications. The aim is to define a practical research agenda for moving AI from algorithm development to responsible implementation in otology and neurotology. For the purposes of this review, otology and neurotology are considered as the clinical continuum encompassing hearing, vestibular, temporal-bone, cochlear-implant, and related skull-base disorders in which AI may contribute directly to diagnosis, monitoring, rehabilitation, procedural planning, or decision support. The review therefore prioritizes applications with a direct audiovestibular or otological translational pathway; adjacent fields, including broader facial-nerve analysis and skull-base AI outside this pathway, are discussed only where they intersect with these core domains. In contrast with recent reviews that primarily catalog available AI applications, the present review specifically evaluates the degree of clinical readiness of each application, emphasizing external validation, prospective evaluation, workflow integration, clinical utility, safety, and regulatory preparedness.

## 2. Literature Search Strategy

A narrative literature review was conducted to identify publications addressing current and emerging applications of AI in otology and neurotology. PubMed/MEDLINE, Scopus, and Web of Science were searched from database inception to 31 July 2026. Search terms included combinations of “artificial intelligence”, “machine learning”, “deep learning”, “neural networks”, “otology”, “neurotology”, “audiology”, “hearing loss”, “hearing screening”, “hearing aids”, “cochlear implant”, “tinnitus”, “vestibular disorders”, “vertigo”, “nystagmus”, “video-oculography”, “temporal bone imaging”, “vestibular schwannoma”, “cholesteatoma”, “robotic surgery”, “wearable sensors”, “telemedicine”, and “digital phenotyping”. Representative combinations included AI-related terms (for example, “artificial intelligence” OR “machine learning” OR “deep learning” OR “neural networks”) combined with domain-specific terms such as “hearing loss”, “hearing screening”, “hearing aids”, “cochlear implant”, “tinnitus”, “vestibular disorders”, “vertigo”, “nystagmus”, “video-oculography”, “temporal bone imaging”, “vestibular schwannoma”, “cholesteatoma”, “robotic surgery”, “wearable sensors”, “telemedicine”, and “digital phenotyping”. Search combinations were adapted to the syntax of each database.

Reference lists of relevant articles were manually screened to identify additional publications. Peer-reviewed original studies, systematic and narrative reviews, meta-analyses, consensus documents, and relevant institutional or regulatory publications written in English were considered. Priority was given to recent clinically relevant studies and to publications describing diagnostic, prognostic, therapeutic, rehabilitative, imaging, surgical, or remote- monitoring applications. Earlier landmark publications were retained when required to provide methodological or historical context.

Studies were selected according to their relevance to the predefined clinical domains and their contribution to understanding clinical applicability, technological development, methodological limitations, or future priorities. Selection was purposive rather than exhaustive and prioritized studies that were most informative for clinical translation. Primary studies providing external, multicenter, prospective, comparative, or clinically relevant validation were given greater weight when judging readiness for implementation, whereas systematic and narrative reviews, consensus documents, and regulatory publications were used primarily to contextualize the field and support cross-cutting methodological or regulatory considerations. Because the review was not designed as a systematic or scoping review, a PRISMA-style record of the number of records identified, screened, excluded, and retained was not prospectively maintained; we therefore do not report a retrospective pseudo-systematic study count.

Because this was a narrative review and the available literature was highly heterogeneous in study design, datasets, algorithms, outcomes, and validation methods, no formal risk-of-bias assessment or quantitative meta-analysis was performed. Evidence was synthesized thematically and critically appraised using five translational dimensions: (1) clinical task definition; (2) representativeness and quality of input data; (3) external and prospective validation; (4) demonstration of clinical utility or workflow benefit; and (5) safety, interpretability, and regulatory readiness. These translational dimensions were also used to inform the qualitative maturity categories reported in Table 1. Maturity was judged at the level of the overall evidence base within each clinical domain rather than assigned to individual studies. Applications were considered relatively more mature when the available evidence included independent or multicenter validation, reproducibility across devices or settings, prospective evaluation, and a clearly defined clinical role. Conversely, applications supported predominantly by retrospective, single-center, internally validated, or exploratory studies were categorized as having lower translational maturity. These categories represent a structured narrative appraisal rather than a validated scoring system or formal risk-of-bias assessment and should therefore not be interpreted as universally applicable grades for all studies within a given field.

## 3. A Translational Framework for Evaluating AI

AI applications in otology and neurotology can be placed along a translational continuum. At the earliest stage are proof-of-concept models developed for narrowly defined retrospective tasks. The next stage requires reproducible technical validation across independent datasets and devices. Clinical readiness begins only when a system is prospectively evaluated in the intended workflow, compared with relevant specialists or standards of care, and shown to improve decisions, efficiency, access, safety, or patient outcomes. This continuum is consistent with broader medical-AI implementation models that distinguish technical development from independent validation, prospective clinical evaluation, and demonstrated impact in the intended workflow [13,15,16].

The same technical task may occupy different positions on this continuum depending on context. Automated segmentation of a vestibular schwannoma for research volumetry has a different risk profile from an algorithm used to recommend treatment. Similarly, automated extraction of nystagmus traces may be relatively mature as a preprocessing tool, whereas autonomous exclusion of stroke in acute vestibular syndrome is a high-risk application requiring substantially stronger evidence. Clinical readiness must therefore be judged by intended use, not by algorithmic performance in isolation. This risk- and intended-use-based interpretation is consistent with contemporary guidance for clinical evaluation and reporting of medical AI [15,16].

Across all applications, evaluation should include discrimination, sensitivity, specificity, calibration, uncertainty, subgroup performance, robustness to device and protocol variation, and analysis of failure cases. Independent external validation is essential, followed by prospective silent testing and impact evaluation when the output may influence clinical care [13,14,17,18,19]. Table 1 summarizes the principal domains, current maturity, and the evidence still required before routine implementation.

## 4. Hearing Assessment and Rehabilitation: Promising Tools with Uneven Clinical Readiness

### 4.1. Automated Audiometry and Hearing Screening

Automated interpretation of pure-tone audiometry is among the most direct applications of ML in audiology. Recent models have classified conductive, sensorineural, and mixed hearing-loss patterns and identified audiometric configurations with encouraging technical performance [20,21]. These systems may support preliminary triage, quality control, and standardized reporting, particularly in high-volume or resource-limited settings. Nevertheless, an audiogram alone does not establish an etiological diagnosis; otoscopy, tympanometry, speech testing, electrophysiology, clinical history, and specialist assessment remain necessary.

AI-assisted processing of otoacoustic emissions and auditory brainstem responses may also improve automated screening and waveform interpretation [22,23]. Potential benefits include artifact detection, reduction in false referrals, and prioritization of cases requiring diagnostic follow-up. However, performance is strongly dependent on acquisition quality and reference standards. A technically accurate screening classifier is clinically useful only when embedded in a reliable pathway for confirmatory testing and early intervention. This is particularly relevant to newborn and population-based hearing-screening programs, in which automated quality control and prioritization could support large-scale pathways while preserving the need for confirmatory diagnostic assessment. Screening performance should therefore be judged not only by sensitivity and specificity, but also by referral yield, missed-case rates, time to diagnostic confirmation, and linkage to early intervention.

### 4.2. Hearing Aids, Tele-Audiology, and Remote Care

Contemporary hearing aids already incorporate adaptive algorithms for environmental classification, noise reduction, directional processing, feedback control, and automatic program selection [5,7]. AI may further personalize these functions by combining acoustic context, device-use patterns, user adjustments, and patient-reported listening difficulty. The clinically relevant endpoint, however, is not algorithmic adaptation itself but sustained improvement in speech understanding, listening effort, participation, adherence, and quality of life. Emerging data-driven approaches may extend this personalization by learning from user preferences and clinical fitting data; primary studies have explored machine-learning-based gain adjustment and neural-network fitting personalization [24,25].

Smartphone-based audiometry and tele-audiology may expand access to hearing care and facilitate follow-up [26,27]. Their reliability depends on transducer calibration, ambient noise, device variability, patient instructions, and digital competence. Remote tools are therefore best positioned as components of structured pathways for screening, monitoring, counseling, or rehabilitation rather than complete replacements for diagnostic audiology.

### 4.3. Cochlear Implantation

Cochlear implantation is a major focus of predictive modeling because postoperative performance varies despite established candidacy criteria. ML models have combined demographic, audiological, clinical, cognitive, electrophysiological, and imaging variables to estimate speech-perception outcomes and identify patients at risk of limited benefit [6,28,29]. Such models could improve counseling and guide rehabilitation intensity, but existing evidence is constrained by cohort size, center-specific practices, inconsistent predictors, and heterogeneous outcome measures.

Imaging-based algorithms can segment cochlear and temporal-bone anatomy, localize electrode contacts, estimate insertion depth, and characterize the electrode-to-modiolus relationship [30,31,32,33,34]. These outputs may support surgical planning and image-guided fitting. Translation will require robust performance across implant models, scanners, anatomical variants, and postoperative artifacts, together with evidence that image-informed programming improves functional outcomes. At present, cochlear-implant AI is best considered a counseling and research-support technology; no predictive or image-guided system has yet demonstrated sufficiently broad prospective benefit to replace established multidisciplinary decision-making.

## 5. Vestibular Medicine: High Diagnostic Potential, High Validation Requirements

### 5.1. Eye-Movement Analysis and Acute Vestibular Syndromes

Video head impulse testing, videonystagmography, and video-oculography generate dense time-series data that are well suited to automated analysis. ML can potentially identify abnormal vestibulo-ocular reflex gain, overt and covert saccades, asymmetries, and nystagmus features while reducing operator-dependent variability [35,36,37,38,39]. DL methods have been developed for pupil tracking, nystagmus extraction, torsional nystagmus recognition, and support of benign paroxysmal positional vertigo diagnosis [37,38,39].

In acute vestibular syndrome, computational models integrating oculomotor and vestibular features may assist differentiation between peripheral vestibulopathy and posterior circulation stroke [10,36]. This is clinically important but high risk: inaccurate reassurance could delay stroke diagnosis. Such tools therefore require prospective validation in representative emergency populations, explicit handling of artifacts and indeterminate recordings, and comparison with trained clinicians. They should not be used as autonomous substitutes for bedside examination, neurological assessment, and indicated neuroimaging. This precaution is consistent with the established role of structured bedside oculomotor and vestibular examination in acute vestibular syndrome, including the HINTS examination when performed by appropriately trained clinicians [40].

### 5.2. Balance, Gait, and Fall Risk

Wearable accelerometers, gyroscopes, and inertial measurement units can quantify gait variability, postural sway, head motion, and activity patterns during laboratory tasks or daily life [41,42,43]. AI-based analysis may support fall-risk stratification, monitoring of chronic vestibular dysfunction, and objective assessment of rehabilitation. Real-world sensing is attractive because brief clinic-based tests may not capture symptom fluctuation or context-dependent instability.

Clinical deployment remains limited by inconsistent sensor placement, nonstandard protocols, differences among devices, and the nonspecific nature of many mobility signals. Reduced activity may reflect vestibular impairment, musculoskeletal disease, fear of falling, depression, environmental barriers, or other comorbidities. Sensor outputs therefore require clinical contextualization rather than disease attribution by algorithm alone.

## 6. Tinnitus: Data-Driven Phenotyping Without a Stable Clinical Biomarker

Tinnitus is particularly suited to multivariable analysis because its burden reflects interactions among auditory characteristics, distress, sleep, attention, mood, cognition, and comorbidity [44,45,46]. Unsupervised clustering has been used to identify tinnitus subgroups, while supervised models have explored diagnosis, severity estimation, and treatment-response prediction [44,45,46,47,48,49]. These approaches may improve stratification beyond a single severity score and may help match patients to counseling, cognitive behavioral therapy, sound-based approaches, or multidisciplinary care.

Digital interventions and smartphone platforms can collect repeated symptom ratings, contextual information, adherence data, and treatment responses [47,48,50]. AI could use these longitudinal data to adapt intervention content or identify early nonresponse. However, app-based improvement should not be conflated with AI efficacy unless the algorithm itself is prospectively evaluated. Engagement bias, attrition, variable therapeutic content, and privacy concerns remain major limitations.

EEG and neuroimaging studies have also used DL and multimodal analysis to search for objective tinnitus-related signatures [51,52,53]. Although these studies are scientifically informative, there is no universally accepted diagnostic biomarker. Models trained in selected research cohorts may capture study-specific acquisition or labeling patterns rather than tinnitus biology. Replication in independent datasets and clinically interpretable endpoints are therefore essential. Consequently, the most realistic near-term role of AI in tinnitus is patient stratification and longitudinal monitoring rather than stand-alone diagnosis or treatment selection.

## 7. Otological Imaging: The Most Technically Mature Domain

### 7.1. Temporal Bone Segmentation and Surgical Planning

DL-based segmentation of temporal-bone CT and cone-beam CT can delineate the cochlea, vestibule, semicircular canals, ossicles, facial nerve, internal auditory canal, and other surgically relevant structures [31,32,33,34]. Automated segmentation may reduce manual workload, improve reproducibility, enable three-dimensional reconstruction, and support patient-specific planning. Multicenter studies are especially valuable because they test robustness across scanners and acquisition protocols [32].

Clinical reliability requires more than high average overlap with manual annotations. Small segmentation errors may be consequential near the facial nerve, labyrinth, carotid canal, or dura. Evaluation should include failure analysis, anatomical variants, disease states, postoperative anatomy, and uncertainty estimation. Human review remains mandatory for safety-critical applications.

### 7.2. Vestibular Schwannoma

MRI-based DL models can automate vestibular schwannoma segmentation and volumetric follow-up [54,55,56]. Automated volumetry could improve consistency during observation and reduce the burden of serial assessment. Radiomic features have also been investigated in relation to hearing status, vascularity, growth, and treatment- related characteristics [55,56].

Before routine use, algorithms must demonstrate robustness across tumor size, cystic change, postoperative or post- radiotherapy appearances, MRI field strength, sequence selection, and institutional protocols. Radiomic associations also require biological plausibility, prespecified analysis, and external validation to avoid overfitting.

### 7.3. Chronic Otitis Media and Cholesteatoma

AI models have been explored for chronic otitis media and cholesteatoma using CT and otoscopic or endoscopic images [57,58]. Potential applications include lesion detection, recognition of erosive changes, and standardized image interpretation. Yet inflammatory tissue, granulation, postoperative change, and cholesteatoma can overlap radiologically. AI output must therefore be integrated with otomicroscopy, audiology, diffusion-weighted MRI when indicated, and surgical judgment. Among otological applications, segmentation and volumetry are closest to routine deployment because the tasks are well defined and directly auditable; diagnostic and treatment-prediction models remain less mature. Otoscopic and endoscopic image classification represents a related, clinically intuitive use case, with primary studies demonstrating automated recognition of tympanic-membrane abnormalities and cholesteatoma from otoendoscopic or otoscopic images [59]. Nevertheless, image quality, illumination, field of view, canal obstruction, postoperative anatomy, and overlap among inflammatory and keratinizing lesions may substantially affect generalizability (Table 2).

**Table 2:** Representative studies and their position along the translational pathway. Studies were purposively selected to illustrate different positions along the translational pathway, with priority given to clinical relevance, methodological and translational informativeness, level of validation, recency where appropriate, and representation of the principal clinical domains covered by the review. The table is illustrative rather than exhaustive.

Reference	Application	Data/Approach	Main Contribution	Translational Appraisal
Kassjański et al. [20]	Audiogram classification	Pure-tone data; supervised classification	Automated hearing-loss type classification	Promising narrow task; requires workflow and external validation
Dou et al. [21]	Audiogram pattern recognition	Deep learning on pure-tone audiograms	Automated classification of audiometric patterns	Dependent on labeling quality and clinical context
Shafieibavani et al. [28]	Cochlear implant outcomes	Multivariable predictive models	Assessed performance, generalizability, and cohort-size effects	Highlights instability of small-cohort models
Ahmadi et al. [10]	Acute vestibular disorders	ML using vestibular/clinical features	Support for central versus peripheral differentiation	High-stakes application needing prospective emergency validation
Lee et al. [37]	Nystagmus extraction	AI analysis of VNG video	Automated extraction of eye-movement signals	Useful technical pipeline; clinical classification still contextual
Mun et al. [39]	BPPV support	DL-based nystagmus detection	Automated recognition relevant to positional vertigo	Needs broad validation across recording conditions
Guillard et al. [45]	Tinnitus subtyping	Comparison of clustering methods	Data-driven multidimensional phenotypes	Exploratory; cluster stability and clinical utility remain uncertain
Wang et al. [32]	Temporal-bone segmentation	Multicenter CT neural network	Automated segmentation across centers	Relatively advanced technical validation; safety review still essential
Łajczak et al. [54]	Vestibular schwannoma segmentation	Systematic review and meta-analysis	Synthesized DL segmentation accuracy	Supports feasibility; heterogeneity limits direct implementation
Caversaccio et al. [60]	Robotic cochlear implantation	Image-guided robotic access	First clinical feasibility experience	Specialized technology; outcome and cost-effectiveness evidence evolving

## 8. AI-Assisted Surgery and Rehabilitation: From Feasibility to Demonstrated Benefit

### 8.1. Robotics, Navigation, and Intraoperative Assistance

Robotic and image-guided systems have been developed to improve trajectory accuracy and reproducibility in cochlear implantation and middle-ear access [60,61,62,63,64]. Patient-specific segmentation, navigation, automated drilling constraints, and augmented-reality visualization may reduce manual variability and support minimally invasive approaches. These technologies are best understood as integrated engineering systems rather than isolated AI algorithms.

Temporal-bone surgery has an exceptionally small margin for error. Consequently, validation must address registration accuracy, mechanical and software failure modes, anatomical deformation, intraoperative updating, emergency override, and surgeon training. Current evidence supports feasibility in selected settings, but broader adoption will depend on comparative clinical outcomes, hearing preservation, procedure time, cost-effectiveness, and scalability.

### 8.2. Auditory and Vestibular Rehabilitation

AI-assisted rehabilitation platforms may adapt auditory or vestibular exercises according to performance, symptoms, adherence, and sensor-derived measures [6,28,29,65,66]. In cochlear implant users, this could support individualized auditory training and remote follow-up. In vestibular rehabilitation, wearable or tablet-based systems can quantify head movements, gaze-stabilization performance, postural control, and completion of home exercises [65,66].

The relevant clinical question is whether adaptation improves adherence, functional recovery, participation, and quality of life compared with well-delivered conventional rehabilitation. Trials should distinguish the effect of the AI component from the effects of increased contact, reminders, gamification, or digital delivery. Translation should therefore be judged by functional outcomes and implementation benefit, not by technical personalization alone.

## 9. Digital Phenotyping and Cognitive Considerations: Emerging but Exploratory Applications

Digital phenotyping uses data generated by smartphones, wearable devices, sensors, and digital interactions to characterize behavior and physiology over time [67,68]. In audiovestibular care, potentially relevant measures include mobility, gait, social interaction, communication patterns, sleep, activity, device use, listening environments, symptom fluctuation, and rehabilitation adherence. These measures may complement episodic clinic visits and support earlier recognition of functional deterioration.

Hearing loss is associated with increased listening effort and is recognized as a potentially modifiable risk factor for cognitive decline [8,9,69]. Vestibular dysfunction may also affect visuospatial processing, attention, memory, and navigation [70,71]. Multimodal monitoring could therefore help investigate relationships among sensory impairment, mobility, social participation, and cognition. However, passive digital measures are indirect and nonspecific; they should not be interpreted as diagnostic biomarkers without validation against clinically meaningful reference standards.

AI-mediated cognitive offloading—the delegation of information retrieval, summarization, planning, or problem solving to AI systems—has been proposed as a potential influence on cognitive engagement [72,73]. This concept is hypothesis-generating rather than established clinical evidence in otology or neurotology. Future longitudinal studies could evaluate whether the intensity and type of AI use interact with hearing status, listening effort, age, education, cognitive reserve, social participation, and hearing rehabilitation. AI may either reduce active cognitive effort or enhance accessibility and engagement, depending on how it is designed and used. The appropriate objective is therefore not to discourage AI use, but to preserve autonomy and active participation while leveraging its assistive benefits. These hypotheses should be presented as a future research direction rather than an established clinical consequence of AI use. More broadly, cognitive-offloading research suggests that external tools can alter how individuals allocate memory, attention, and problem-solving effort, although the direction and clinical significance of these effects depend on task demands, user characteristics, and the way the technology is integrated into daily activity. Evidence specifically linking generative-AI use to hearing, vestibular function, or audiovestibular rehabilitation remains limited, and this proposed relationship should therefore be regarded as a conceptual research hypothesis rather than a demonstrated clinical effect. This interpretation is supported by broader cognitive-offloading literature and emerging studies of AI-mediated cognition, while evidence of direct audiovestibular effects remains absent [73,74,75].

## 10. Cross-Cutting Barriers to Clinical Translation

### 10.1. Dataset Quality, Bias, and Generalizability

Many AI studies in otology and neurotology use small, retrospective, single-center, or demographically selected datasets [13,14]. Models may learn center-specific acquisition patterns, referral pathways, device characteristics, or annotation conventions rather than generalizable disease features. Dataset shift is especially relevant when algorithms are transferred across audiometers, hearing devices, VOG/VNG systems, scanners, languages, and healthcare systems. Development datasets should therefore represent the intended population, and performance should be reported across clinically relevant subgroups.

### 10.2. Validation, Reporting, and Clinical Utility

Internal cross-validation is insufficient for deployment. Independent external validation, prospective silent evaluation, and impact studies are needed to determine whether AI improves decisions or outcomes. Reporting should include cohort assembly, missing-data handling, reference standards, preprocessing, model specification, calibration, confidence intervals, subgroup analyses, and failure cases. CONSORT-AI, SPIRIT-AI, and TRIPOD + AI provide relevant reporting frameworks for trials, protocols, and prediction models [17,18,19]. DECIDE-AI, which addresses early-stage prospective clinical evaluation of AI decision-support systems, and the updated CLAIM guideline, which focuses on reporting of AI applications in medical imaging, provide complementary guidance [15,16].

Direct comparison with trained clinicians or the current standard of care is especially important when an AI output is intended to influence diagnosis or management. At present, such head-to-head prospective evaluations remain limited in otology and neurotology, and many studies instead report algorithmic performance against retrospective labels or technical reference standards. Interoperability should likewise be treated as an empirical validation target: performance should be tested across devices, manufacturers, software environments, acquisition protocols, and data formats rather than assumed from single-platform development.

### 10.3. Explainability and Human Oversight

Explainability can support error detection, clinician trust, patient communication, and regulatory review, but post hoc visualizations should not be assumed to reveal causal reasoning. The level of explanation required depends on risk and intended use. A low-risk triage aid may tolerate less interpretability than a model influencing stroke exclusion, implant candidacy, surgical trajectory, or autonomous intervention. In all high-risk applications, clinicians must be able to reject or override the output and understand the conditions under which the system may fail.

### 10.4. Privacy, Regulation, and Accountability

Audiological, vestibular, imaging, behavioral, and wearable data may be sensitive and longitudinally identifiable. Data minimization, secure storage, access control, transparent consent, cybersecurity, and governance of secondary use are essential [12,13,14,76,77,78]. In Europe, software may fall under the Medical Device Regulation, while the AI Act establishes a risk-based framework relevant to high-risk medical AI [77,78]. Responsibility for errors should be explicitly allocated among clinicians, institutions, developers, and manufacturers; deployment should include monitoring for performance drift and mechanisms for incident reporting. In the European setting, these requirements should also be interpreted in conjunction with the General Data Protection Regulation (GDPR), particularly where longitudinal, biometric, behavioral, or remotely collected patient data are processed [79]. Regulatory status should be described at the level of the intended use of the specific software, since the same underlying AI technique may fall into different risk categories depending on whether it performs low-risk workflow support, diagnostic assistance, treatment recommendation, or safety-critical intervention. As a concrete deployment example, the RobOtol^®^ platform (Collin Medical, Bagneux, France) has been used clinically for robot-assisted cochlear-implant electrode insertion; this illustrates that clinical deployment may occur at the level of an integrated device even when the system is not itself an autonomous AI decision-maker [80] (Table 3).

**Table 3:** Minimum evidence required before routine clinical deployment of AI systems.

Requirement	Why It Matters	Recommended Evidence Before Routine Use
Representative data	Reduces spectrum and demographic bias	Multicenter datasets reflecting intended patients, devices, and settings
External validation	Tests generalizability beyond development data	Independent temporal and geographic validation
Prospective evaluation	Detects workflow and implementation effects	Silent prospective study followed by impact evaluation
Calibration and uncertainty	Supports safe interpretation of predicted risk	Calibration plots/metrics and explicit indeterminate outputs
Clinical comparator	Establishes added value	Comparison with specialists and current standard of care
Subgroup and fairness analysis	Identifies unequal performance	Results by age, sex, disease severity, device, and relevant population groups
Explainability and failure analysis	Supports oversight and error detection	Clinically meaningful explanations and documented failure modes
Interoperability	Enables reproducible workflow integration	Testing across systems, manufacturers, and data formats
Privacy and cybersecurity	Protects sensitive longitudinal data	Data governance, security testing, and compliant consent
Post-deployment monitoring	Identifies drift and rare harms	Performance surveillance, audit trails, update control, and incident reporting

## 11. A Research Agenda for Clinically Responsible AI

The next phase of research should be organized around intended clinical use rather than model novelty. Priority should be given to tasks with a clear unmet need, a measurable clinical consequence, and a realistic path to validation. Development studies should predefine the target population, workflow, comparator, decision threshold, and clinically meaningful endpoint before model training begins.

Multicenter collaboration is essential for reducing center-specific bias and testing performance across devices, languages, acquisition protocols, and healthcare systems. Prospective silent deployment can identify workflow failures without influencing care, after which controlled impact studies should determine whether the system improves outcomes, efficiency, access, or safety. Federated and privacy-preserving methods may facilitate data sharing, but they cannot substitute for harmonized labeling and clinically valid reference standards. Such studies should deliberately incorporate cross-device and cross-platform testing and, where AI influences a clinical decision, prespecified comparison with appropriately trained clinicians or current standard-of-care pathways.

Priority areas include cross-device validation of automated eye-movement analysis, prospective AI-assisted triage in acute vestibular syndrome, clinically meaningful prediction of cochlear-implant outcomes, image-informed fitting evaluated against patient benefit, longitudinal digital phenotyping, adaptive rehabilitation trials, and cost-effectiveness assessment of robotic systems. Generative AI and large language models may assist documentation, education, and knowledge retrieval, but patient-facing or safety-critical use requires domain-specific evaluation, source traceability, and mandatory human verification.

## 12. Conclusions

AI in otology and neurotology has moved beyond technical novelty, but not yet beyond the translational gap.

Narrow classification, signal-extraction, segmentation, and volumetric tasks are the most mature because their outputs are relatively well defined and auditable. Multimodal prognosis, treatment selection, digital phenotyping, autonomous decision support, and AI-assisted surgery remain earlier in development. The decisive evidence for adoption will not be a higher accuracy value, but reproducible improvement in clinical decisions, patient outcomes, safety, accessibility, or workflow efficiency. Responsible implementation requires representative data, external and prospective validation, calibrated uncertainty, clinically meaningful comparators, interoperability, transparent human oversight, regulatory and privacy compliance, and post-deployment surveillance. AI should therefore be implemented as an auditable, human-supervised technology that augments specialist judgment rather than replacing it.

## Figures and Tables

**Table 1:** Translational maturity of principal AI applications in otology and neurotology. Translational maturity reflects the authors’ structured qualitative appraisal of the overall evidence base within each domain according to the five dimensions defined in Section 2; it is not a validated grading system for individual studies.

Clinical Domain	Representative AI Task	Current Translational Maturity	Most Realistic Near-term Role	Evidence Still Required
Hearing assessment	Audiogram classification; OAE/ABR interpretation	Moderate for narrow classification tasks	Triage, quality control, standardized reporting	External device-level validation and prospective workflow impact
Hearing aids and remote care	Adaptive processing; benefit prediction; tele-audiology	Moderate for embedded signal processing; low-to-moderate for clinical prediction	Personalized fitting and structured remote follow-up	Patient-centered outcome trials, calibration, and equity analysis
Cochlear implantation	Outcome prediction; electrode localization; image-guided fitting	Moderate for segmentation/localization; low for outcome-guided decisions	Counseling, planning, and research-supported programming	Large multicenter cohorts and prospective demonstration of functional benefit
Vestibular medicine	Nystagmus extraction; vHIT analysis; central/peripheral classification	Moderate for signal extraction; low for high-stakes diagnosis	Automated preprocessing and specialist decision support	Cross-device validation and prospective emergency-setting studies
Tinnitus	Phenotyping; clustering; response prediction	Low-to-moderate	Patient stratification and longitudinal monitoring	Stable phenotypes, reproducible biomarkers, and treatment-impact validation
Otological imaging	Temporal-bone and tumor segmentation; volumetry	Moderate-to-high for segmentation and volumetry	Workflow acceleration, planning, and reproducible follow-up	Robustness across scanners/pathology and safety-oriented validation
Robotics and surgery	Trajectory planning; navigation; landmark recognition	Low-to-moderate	Specialist assistance in highly selected procedures	Comparative outcomes, cost-effectiveness, and failure-mode evaluation
Digital phenotyping	Mobility, balance, communication, and symptom monitoring	Low/exploratory	Longitudinal functional monitoring	Specificity, adherence, privacy safeguards, and clinical-action thresholds

## Data Availability

No new data were created or analyzed in this study. Data sharing is not applicable to this article.
